# Suppression of Deactivation of Working Memory and Promotion of Activation of Sustained Attention in the Default Mode Network Are Affected by Schizotypy in a Large Sample of Nonclinical Subjects

**DOI:** 10.1002/brb3.70449

**Published:** 2025-05-07

**Authors:** Seishu Nakagawa, Hikaru Takeuchi, Yasuyuki Taki, Ryoichi Yokoyama, Kohei Sakaki, Kelssy Hitomi dos Santos Kawata, Takayuki Nozawa, Susumu Yokota, Tsuyoshi Araki, Rui Nouchi, Ryuta Kawashima

**Affiliations:** ^1^ Division of Psychiatry Tohoku Medical and Pharmaceutical University Sendai Japan; ^2^ Department of Human Brain Science, Institute of Development, Aging and Cancer Tohoku University Sendai Japan; ^3^ Division of Developmental Cognitive Neuroscience, Institute of Development, Aging and Cancer Tohoku University Sendai Japan; ^4^ Division of Medical Neuroimage Analysis, Department of Community Medical Supports, Tohoku Medical Megabank Organization Tohoku University Sendai Japan; ^5^ Department of Nuclear Medicine and Radiology, Institute of Development, Aging and Cancer Tohoku University Sendai Japan; ^6^ Department of Extended Intelligence for Medicine, The Ishii‐Ishibashi Laboratory Keio University Tokyo Japan; ^7^ Department of Advanced Brain Science, Institute of Development, Aging and Cancer Tohoku University Sendai Japan; ^8^ Graduate School of Medicine The University of Tokyo Tokyo Japan; ^9^ Graduate School of Interdisciplinary Information Studies The University of Tokyo Tokyo Japan; ^10^ Faculty of Engineering University of Toyama Toyama Japan; ^11^ Faculty of Arts and Science Kyushu University Fukuoka Japan; ^12^ ADVANTAGE Risk Management Co., Ltd. Tokyo Japan; ^13^ Creative Interdisciplinary Research Division, Frontier Research Institute for Interdisciplinary Science (FRIS) Tohoku University Sendai Japan; ^14^ Smart Aging International Research Center, Institute of Development, Aging and Cancer Tohoku University Sendai Japan; ^15^ Human and Social Response Research Division, International Research Institute of Disaster Science Tohoku University Sendai Japan

**Keywords:** default mode network, schizotypy, sustained attention, working memory

## Abstract

**Introduction:**

Schizotypy is a personality trait characterized by subclinical expression of the signs and symptoms of schizophrenia. Maladaptive schizotypy is given the clinical designation of schizotypal personality disorder. Previous studies have shown that functional disturbance of the default mode network (DMN) is associated with maladaptive schizotypy. Working memory impairment is a particularly common feature of maladaptive schizotypy and schizophrenia. As these characteristics can also be observed in the healthy population, our purpose was to identify the neural correlates of schizotypy during sustained attention tasks and working memory tasks in nonclinical subjects.

**Methods:**

We recruited 409 healthy individuals (251 men and 158 women; 20.7 ± 1.8 [SD] years of age) and determined their schizotypy scores using the Schizotypal Personality Questionnaire. We used a typical functional MRI (fMRI) task under 0‐back and 2‐back conditions. Corrections for multiple comparisons were performed via the threshold‐free cluster enhancement method with a familywise error‐corrected threshold of *p* < 0.0167 (0.05/3) at the whole‐brain level, including the cerebellum.

**Results:**

Suppression of deactivation of the DMN (the medial prefrontal cortex (mPFC), the posterior cingulate cortex, the inferior parietal lobule, and the middle temporal gyrus) in the working memory (2‐back > rest) task and promotion of activation during the sustained attention (0‐back > rest) task were associated with schizotypy scores. Among the schizotypy subscores, cognitive–perceptual deficits were significantly related only to the left precuneus, the mPFC, and the superior temporal gyrus for the 2‐back > 0‐back contrast; to the left precuneus and the bilateral mPFC for the 2‐back > rest contrast; and to the left superior temporal cortex and the right precuneus for the 0‐back > rest contrast within the DMN.

**Conclusion:**

Disturbance of the DMN is related to the degree of schizotypy, especially to the degree of cognitive‒perceptual deficits, even in nonclinical subjects.

## Graphical abstract

The greater the schizotypy is, the weaker task‐induced deactivation of the default mode network is during the task.

The observed cognitive–perceptual deficits were mainly related to disturbances in the default mode network.

Disturbance of the default mode network might be a common feature in both the nonclinical and the clinical schizophrenia spectra.

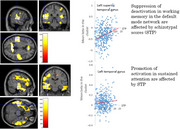

## 1 Introduction

Schizotypy (schizophrenic phenotype) is a personality trait that is characterized by the subclinical expression of the signs and symptoms of schizophrenia (Ettinger et al. 2015). Maladaptive schizotypy is given the clinical designation of schizotypal personality disorder (Rivera Tapia 2022). Schizotypy in healthy populations and the schizophrenia spectrum appear to be linked, as supported by social and environmental evidence (Nelson et al. 2013). Moreover, schizotypy is related to an increased risk of schizophrenia spectrum disorders (Barrantes‐Vidal et al. 2020) and schizophrenia (K. K.‐Y. Wong and Raine 2020). Importantly, the World Health Organization classifies schizotypal personality disorder as a form of schizophrenia in the *International Classification of Diseases, 11th Revision (ICD‐11)* (Bach and First 2018). On the other hand, according to the *Diagnostic and Statistical Manual of Mental Disorders, 5th Edition, Text Revision (DSM‐5‐TR)* (Francois and Torrico 2024), the course of schizotypal personality disorder is relatively stable; few individuals with the disorder develop schizophrenia.

Although there are overlapping findings concerning brain function in schizophrenia patients and individuals with clinically maladaptive schizotypy, the idea that the neural correlates of schizotypy in nonclinical individuals overlap with those of clinical schizophrenia patients is controversial (Ettinger et al. 2015).

There are numerous conflicting findings showing deactivation and activation within the default mode network (DMN) in patients with schizophrenia (Hu et al. 2017). The DMN, which shows decreased activation during goal‐oriented or attention‐demanding tasks, is modulated by cognitive load (Fransson 2006). Furthermore, functional changes in the DMN, specifically in the frontal and temporal cortices, have been associated with schizotypy (Tonini et al. 2021). One review revealed increased task‐related activity in frontal cortex regions in individuals with a high degree of schizotypy (Kozhuharova et al. 2020). Interestingly, not only patients with schizophrenia but also their relatives showed reduced visual working memory task‐related suppression in the medial prefrontal cortex (mPFC) (Whitfield‐Gabrieli et al. 2009). Dysfunction of brain circuits with dysregulation of dopaminergic pathways is less severe in maladaptive schizotypy than in schizophrenia (Attademo et al. 2021).

With respect to cognitive function, schizotypy is related to lower performance in areas related to global attention and processing speed (Siddi et al. 2017), sustained attention, and memory (Ettinger et al. 2015). Impairments of visual working memory are common features of psychiatric disorders (Wang et al. 2021), especially schizotypy and schizophrenia, and large effect sizes are observed (Siddi et al. 2017). Intact discrimination of form and trajectory is impaired during visual working memory tasks in patients with maladaptive schizotypy (Farmer et al. 2000). A strong functional magnetic resonance imaging (fMRI) response in the right mPFC and bilateral anterior cingulate cortex during visual working memory tasks in patients with schizophrenia has been observed (Wang et al. 2021). With respect to sustained attention, decreased task‐induced activation of the left postcentral gyrus in a 0‐back (sustained attention) task was detected in a study in which 15 individuals with maladaptive schizotypy were compared to 16 healthy controls using the Structured Clinical Interview for SDM‐IV‐TR Axis I (SCID) and Axis II Disorders SCID II (Vu et al. 2013). Furthermore, schizotypal traits in nonclinical subjects appear to be associated with cognitive deficits similar to those seen in patients with schizophrenia but with attenuated severity (Siddi et al. 2017). Task‐induced deactivation of the DMN during sustained attention and visual working memory tasks can be disturbed by trends of schizotypy, although consistent patterns have not been observed due to methodological differences (Tonini et al. 2021).

Working memory capacity is necessary for daily living, and many real‐world phenomena are explained by differences in individual abilities in visual working memory function (Draheim et al. 2022). However, to our knowledge, no study has evaluated the neural correlates of schizotypy in a large sample of nonclinical individuals during the performance of cognitive tasks with high attentional demands.

Our purpose in this study was to ascertain, by testing a large sample of nonclinical subjects using standard sustained attention and working memory tasks, whether the DMN of individuals with nonclinical schizotypy is disturbed.

## 2 Subjects and Methods

The present study, which is a part of an ongoing project in which associations among brain imaging, cognitive function, and aging are being investigated, included 409 healthy right‐handed individuals (251 men and 158 women) from whom the data necessary for whole‐brain (including cerebellum) analyses were obtained. The mean age of the subjects was 20.7 years (standard deviation [SD], 1.8; range, 18–27 years). The subjects were university students, postgraduates, or university graduates who had graduated within the past year. All the subjects had normal vision and no history of neurological or psychiatric illness. Thus, no individuals with maladaptive schizotypy were included in this study.

### 2.1 Psychological Measures

Schizotypy has three components: cognitive‒perceptual deficits, interpersonal deficits, and disorganization (Wong and Raine 2018). We used the Japanese version of the Schizotypal Personality Questionnaire (SPQ), which consists of 37 items translated from the original version by Raine (1991) into Japanese (Iijima et al. 2010). The response format was “yes/no.” All items answered “yes” were assigned one point. The SPQ has high sampling, convergent, and discriminant validity and high internal reliability and test–retest reliability (Iijima et al. 2010; Raine 1991). The questionnaire incorporates a three‐factor model reflecting cognitive–perceptual factors, for example, “Have you ever seen things invisible to other people?”; interpersonal factors, for example, “I am sure I am being talked about behind my back”; and disorganized latent factors, for example, “People sometimes find it hard to understand what I am saying” (Raine et al. 1994).

Raven's advanced progressive matrix (RAPM) (Raven 2000) is a nonverbal reasoning task and a representative measure of general intelligence. For further details, please see the Supporting Information.

### 2.2 fMRI Task

fMRI was used to map brain activity during the performance of cognitive tasks. The descriptions of the tasks are mostly reproduced from a previous publication in which the same methods were used (Takeuchi et al. 2015). The *n*‐back task is a typical fMRI task with 0‐back (simple cognitive process) and 2‐back (working memory) conditions. We used a simple block design.

The inverted U‐shaped activity patterns that are observed due to the *n*‐back task's load and activity patterns (namely, the activity increases as the load increases, but when the tasks are very difficult, the activity decreases) (Callicott et al. 2003) are not expected to have an effect in this study because the average accuracy rates for the 2‐back task are almost 100%. For further details, please see the Supporting Information.

### 2.3 Image Acquisition

The MRI acquisition methods have been described previously (Takeuchi et al. 2012), and the relevant text is reproduced below. All MRI data acquisition was performed using a 3‐T Philips Achieva scanner. Forty‐two transaxial gradient‐echo images (TR = 2.5 s, TE = 30 ms, flip angle = 90°, slice thickness = 3 mm, FOV = 192 mm, matrix = 64 × 64) covering the entire brain were acquired via an echo planar sequence.

For more details, please see the Supporting Information.

### 2.4 Preprocessing of the Task‐Based fMRI Data

Preprocessing and analysis of functional activation data were performed via SPM12 implemented in MATLAB. Most of the following descriptions are reproduced from our previous study (Takeuchi et al. 2015), in which similar methods were used. Before the analysis, blood oxygen level–dependent (BOLD) images were realigned and resliced to the mean of the BOLD images, which was then realigned to the mean *b* = 0 image as previously described (Takeuchi et al. 2011). The voxel size of the normalized BOLD images was 3 × 3 × 3 mm^3^ (Takeuchi et al. 2015).

### 2.5 First‐Level Analysis of Functional Imaging Data

Individual‐level statistical analyses were performed using a general linear model. A design matrix was fitted to each participant with one regressor in each task condition (0‐ or 2‐back in the *n*‐back task) via the standard hemodynamic response function. After estimation, beta images of contrasts of 2‐back > rest and 0‐back > rest were smoothed (8 mm full‐width half‐maximum) and taken for the second‐level analyses. For additional details, please see the Supporting Information.

### 2.6 Second‐Level fMRI Analyses

The maps of the dependent variables were beta estimate images of 2‐back > 0‐back, 2‐back > rest, and 0‐back > rest contrasts. The covariates included age, sex, RAPM score, accuracy, and reaction time in the 0‐back and 2‐back tasks and volume‐level mean framewise displacement during the scan for the *n*‐back task (Power et al. 2012).

We conducted whole‐brain multiple regression analyses related to the total schizotypy score or to each subscore (cognitive‒perceptual deficits, interpersonal deficits, and disorganization) via three contrasts made via first‐level analyses.

Correction for multiple comparisons was performed via threshold‐free cluster enhancement (TFCE) (Smith and Nichols 2009) with randomized nonparametric testing (5000 permutations) implemented using tools in the TFCE toolbox (https://dbm.neuro.uni‐jena.de/tfce/). The permutation test is one type of nonparametric test that can be used when the assumptions required for a parametric approach are not met (Nichols and Holmes 2002). The familywise error (FWE) threshold was corrected at *p* < 0.05/3, reflecting the three comparisons.

### 2.7 Ethics Statement

All procedures involving human participants were performed in accordance with the ethical standards of the institutional and/or national research committee and with the principles set forth in the 1964 Declaration of Helsinki and its later amendments or comparable ethical standards.

### 2.8 Ethical Approval

Written informed consent was obtained from the adult subjects. For nonadult subjects (age < 20 years), written informed consent was obtained from their parents (guardians). This study was approved by the Ethics Committee of Tohoku University.

## 3 Results

### 3.1 Psychological Results

**FIGURE 1:**
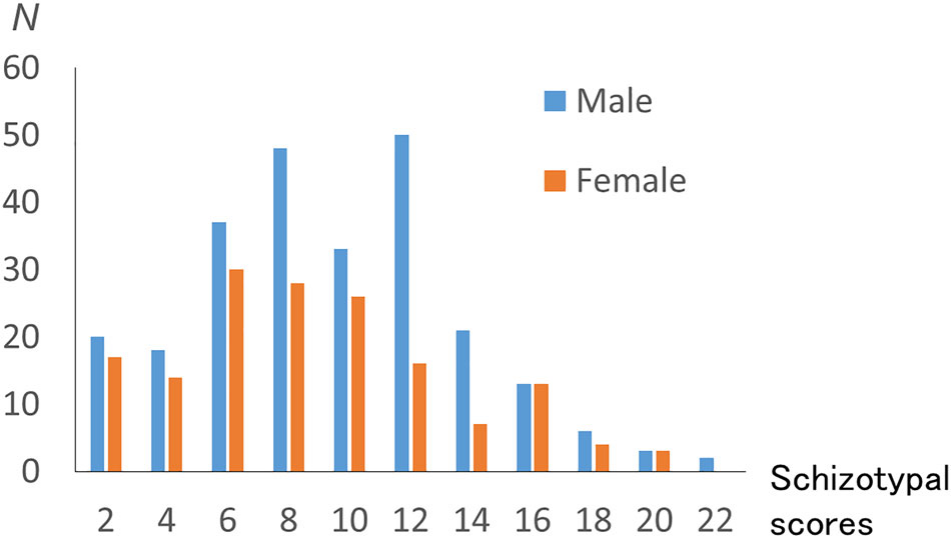
Distribution of total schizotypy scores for males and females. The histograms show the distributions of schizotypy scores in males and females.

The means and SDs of the values obtained for age, general intelligence, and total schizotypy score are presented in Table 1. The distributions of the total schizotypy score and each subscore for males and females are presented in Figure 1.

**TABLE 1:** Sex differences in age, RAPM score, cognitive–perceptual deficits score, interpersonal deficits score, disorganization score, and schizotypy score (mean ± SD) determined via two‐sample *t‐*tests.

Measure	Total	Males	Females	*p*	*t*
Age (years)	20.7 (1.8)	20.8 (1.9)	20.6 (1.6)	0.253	1.1
RAPM score	28.4 (4.0)	28.8 (3.8)	27.7 (4.1)	0.005^*^	2.8
Cognitive–perceptual deficits score	2.0 (2.1)	2.1 (2.0)	1.8 (2.2)	0.158	1.4
Interpersonal deficits score	3.9 (2.0)	4.0 (1.9)	3.8 (2.1)	0.222	1.2
Disorganization score	2.7 (1.7)	2.7 (1.8)	2.6 (1.7)	0.662	0.4
Total schizotypy score	8.6 (4.4)	8.9 (4.3)	8.3 (4.5)	0.164	1.4

Abbreviations: RAPM, Raven's advanced progressive matrix; SD, standard deviation.

* *p* < 0.01.

### 3.2 fMRI Results

#### 3.2.1 2‐Back > 0‐Back Contrast

No significant brain activity related to the total schizotypy score was detected for the “2‐back > 0‐back” contrast via whole‐brain multiple regression analysis.

With respect to the subscores, only the cognitive–perceptual deficits subscore was significantly correlated with beta estimates for the “2‐back > 0‐back” contrast in the bilateral cuneus, the precuneus, the mPFC, the left fusiform gyrus, the inferior temporal gyrus, the right superior temporal gyrus, the central operculum, and the posterior insula (Table 2, Figure 4a1).

**TABLE 2:** Brain regions exhibiting a significant interaction effect between the cognitive–perceptual deficits subscore and the 2‐back > 0‐back contrast on brain activity.

Brain region	R/L	*X*	*Y*	*Z*	TFCE value	Corrected *p‐*value (FWE)	Cluster size (*k*_E_)
Cuneus	L	−6	−69	18	2084	< 0.001	2472
Medial prefrontal gyrus	L	−3	54	−9	1218	0.001	2235
Fusiform gyrus	L	−30	−36	−21	1018	0.002	389
Superior temporal gyrus	R	63	−3	3	997	0.003	482
Central operculum	R	54	−6	−39	960	0.003	756
Inferior temporal gyrus	L	−51	−6	−39	926	0.004	584
Posterior insula	R	21	−9	54	729	0.014	53

Abbreviations: FWE: familywise error, TFCE: threshold‐free cluster enhancement.

*p* < 0.05/3, FWE‐corrected.

#### 3.2.2 2‐Back < 0‐Back Contrast

No significant positive or negative brain activity related to the total schizotypy score was detected for the “2‐back < 0‐back” contrast via whole‐brain multiple regression analysis.

In contrast, the cognitive–perceptual deficits subscore was significantly correlated with beta estimates for the “2‐back < 0‐back” contrast in the bilateral superior parietal gyrus, the left posterior cingulate cortex (PCC), the superior frontal gyrus, the precentral gyrus, the supramarginal gyrus, and the right middle frontal gyrus (Table 3, Figure 4a2).

**TABLE 3:** Brain regions exhibiting significant interaction effects between the cognitive–perceptual deficits subscore and the 2‐back < 0‐back contrast on brain activity.

Brain region	R/L	*X*	*Y*	*Z*	TFCE value	Corrected *p‐*value (FWE)	Cluster size (*k*_E_)
Posterior cingulate cortex	L	−3	−39	12	1360	< 0.001	260
Superior frontal gyrus	L	−6	3	63	1353	< 0.001	426
Middle frontal gyrus	R	33	0	66	995	0.002	150
Superior parietal gyrus	L	−12	−78	51	985	0.002	98
Superior parietal gyrus	R	30	−75	54	947	0.003	102
Precentral gyrus	L	−42	3	30	731	0.012	30
Supramarginal gyrus	L	−45	−48	45	697	0.015	11

Abbreviations: FWE: familywise error, TFCE: threshold‐free cluster enhancement.

*p* < 0.05/3, FWE‐corrected.

#### 3.2.3 2‐Back > Rest Contrast

**FIGURE 2:**
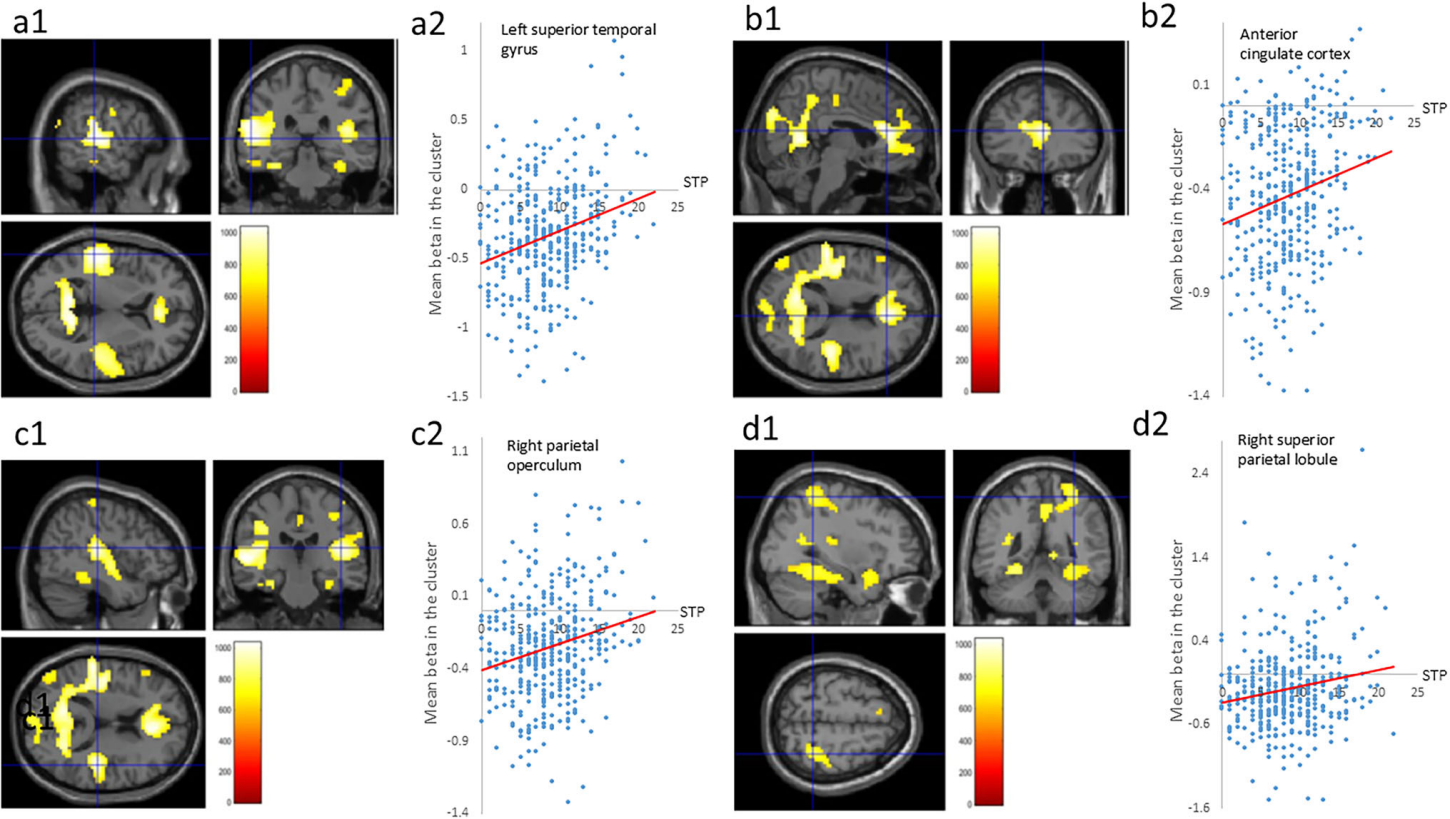
Associations between brain activity during the working memory task and schizotypy scores. Suppression of deactivation in the left superior temporal gyrus (a1), the bilateral anterior cingulate cortex (b1), the right parietal operculum (c1), and the right superior parietal lobule (d1) is associated with schizotypy score during the 2‐back task. The results were obtained using a significance threshold of *p* < 0.05/3, corrected for multiple comparisons based on 5000 permutations using threshold‐free cluster enhancement (TFCE) scores. All the results are overlaid on a “single‐subject T1” SPM12 image. The scatter plots show the associations between each cluster and the eigenvector values of the beta estimates in each cluster (a2, b2, c2, and d2).

Whole‐brain multiple regression analysis revealed that the total schizotypy score was significantly correlated with beta estimates for the “2‐back task > rest” contrast in the area from the left superior temporal gyrus to the inferior parietal lobule (Figure 2a), the area from the anterior cingulate cortex to the bilateral ventral mPFC/medial orbitofrontal cortex (Figure 2b), the right parietal operculum (including the insula) (Figure 2c), the right superior parietal lobule (Figure 2d), the left middle occipital gyrus, the right middle cingulate cortex, the left inferior/middle temporal gyri, and the left inferior/superior frontal gyri (Table 4). Because the regions shown in Figure 2a,b are usually regarded as regions in which task‐induced deactivation occurs (i.e., as DMN regions), higher schizotypy scores led to weaker task‐induced deactivation.

**TABLE 4:** Brain regions exhibiting significant interaction effects between the schizotypy score and the 2‐back > rest contrast on brain activity.

Brain region	R/L	*X*	*Y*	*Z*	TFCE value	Corrected *p‐*value (FWE)	Cluster size (*k*_E_)
Superior temporal gyrus	L	−57	−30	6	1037	0.001	2785
Superior temporal gyrus	L	−54	−21	3	1024	0.001	
Posterior cingulate cortex	L	−9	−54	3	1016	0.001	
Anterior cingulate cortex	R	6	33	15	1008	0.001	645
Anterior cingulate cortex	L	−6	27	18	934	0.002	
Anterior cingulate cortex	L	−12	33	18	932	0.002	
Parietal operculum	R	45	−24	15	976	0.001	1113
Central operculum	R	57	−12	3	871	0.003	
Posterior insula	R	42	−12	−6	848	0.003	
Superior parietal lobule	R	33	−45	60	806	0.004	333
Postcentral gyrus	R	42	−33	66	741	0.007	
Postcentral gyrus	R	39	−24	48	698	0.01	
Middle occipital gyrus	L	−48	−75	21	740	0.007	165
Middle occipital gyrus	L	−45	−81	30	712	0.009	
Middle cingulate cortex	R	3	−21	45	670	0.012	38
Inferior temporal gyrus	L	−51	−30	−18	643	0.015	20
Inferior temporal gyrus	R	51	−6	−42	643	0/015	16
Inferior frontal gyrus	L	−51	27	15	643	0.015	10
Superior frontal gyrus	L	−12	27	54	637	0.016	9
Middle temporal gyrus	L	−51	−6	−24	637	0.016	9

Abbreviations: FWE: familywise error, TFCE: threshold‐free cluster enhancement.

*p* < 0.05/3, FWE‐corrected.

The cognitive–perceptual deficits subscore was significantly correlated with beta estimates for the “2‐back > rest” contrast in the bilateral precuneus, the anterior cingulate cortex, and the cerebellum (Table 5, Figure 4b1).

**TABLE 5:** Brain regions exhibiting a significant interaction effect between the cognitive–perceptual deficits subscore and the 2‐back > rest contrast on brain activity.

Brain region	R/L	*X*	*Y*	*Z*	TFCE value	Corrected *p‐*value (FWE)	Cluster size (*k*_E_)
Precuneus	L	−12	−57	6	2729	< 0.001	15691
Precuneus	L	−9	−66	18	2635	< 0.001	
Anterior cingulate cortex	R	6	33	12	2569	< 0.001	
Cerebellum	R	18	−54	−45	749	0.011	252

Abbreviations: FWE: familywise error, TFCE: threshold‐free cluster enhancement.

*p* < 0.05/3, FWE‐corrected.

#### 3.2.4 2‐Back < Rest Contrast

No significant positive or negative brain activity related to the total schizotypy score was detected for the “2‐back < rest” contrast via whole‐brain multiple regression analysis.

However, the cognitive–perceptual deficits subscore was significantly correlated with beta estimates for the “2‐back < rest” contrast in the bilateral supplementary motor cortex, the precentral cortex, the PC, the left superior parietal cortex, and the right thalamus (Table 6, Figure 4b2).

**TABLE 6:** Brain regions exhibiting a significant interaction effect between the cognitive‒perceptual deficits subscore and the 2‐back < rest contrast on brain activity.

Brain region	R/L	*X*	*Y*	*Z*	TFCE value	Corrected *p‐*value (FWE)	Cluster size (*k*_E_)
Supplementary motor cortex	L	−6	0	60	1919	< 0.001	1681
Precentral gyrus	R	30	−90	−12	1670	< 0.001	1813
Posterior cingulate cortex	L	−12	−42	12	1658	< 0.001	376
Superior parietal cortex	L	−12	−78	51	691	0.013	15
Thalamus	R	15	−18	18	659	0.016	9

Abbreviations: FWE: familywise error, TFCE: threshold‐free cluster enhancement.

*p* < 0.05/3, FWE‐corrected.

#### 3.2.5 0‐Back > Rest Contrast

**FIGURE 3:**
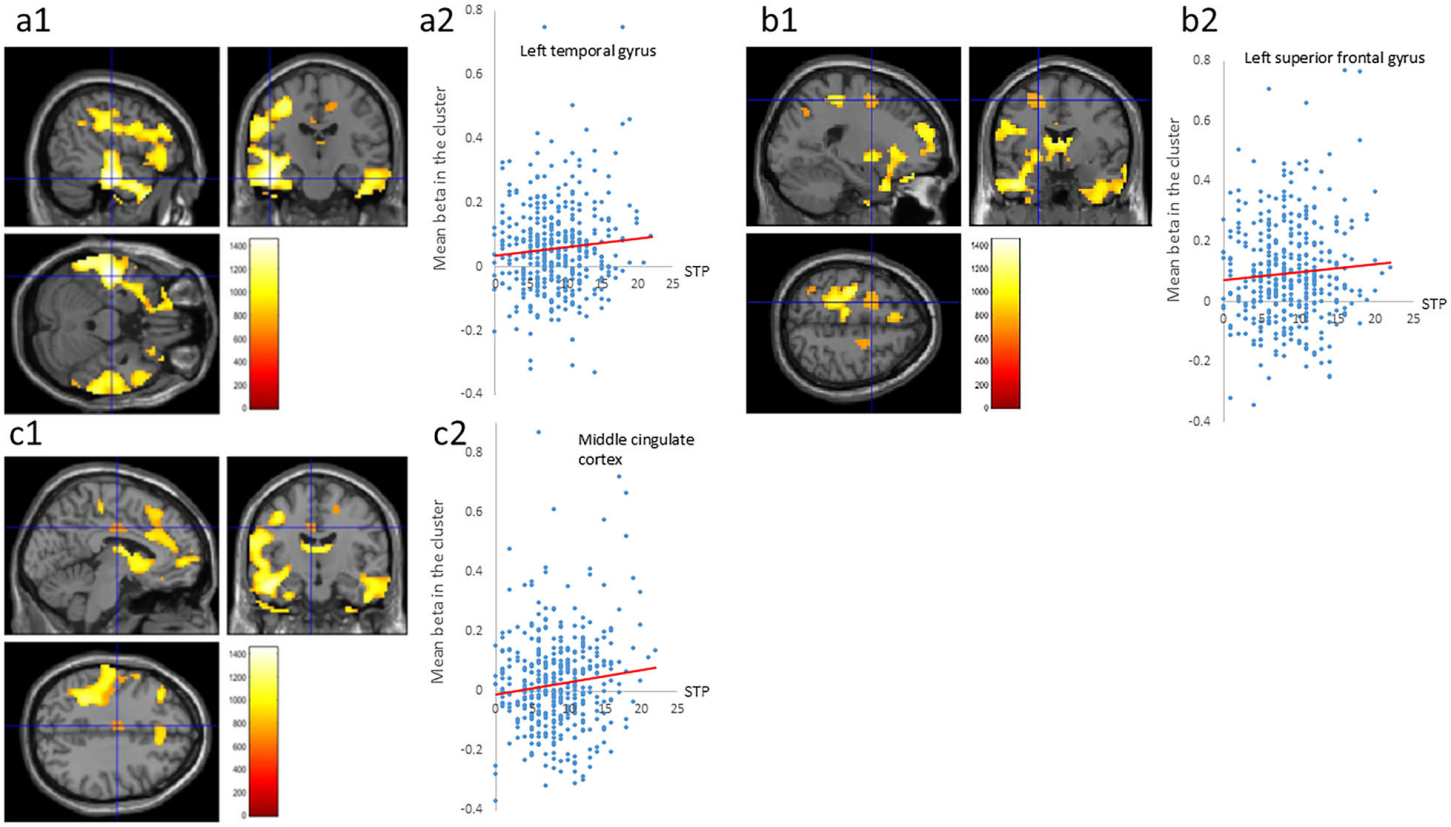
Associations between brain activity during the sustained attention task and schizotypy scores. Brain suppression of activation in the inferior cortex via the middle to superior temporal gyri (a1), the bilateral superior frontal gyri (b1), and the right middle cingulate cortex (c1) during the 0‐back task was associated with schizotypy score. The results were obtained using a significance threshold of *p* < 0.05/3, corrected for multiple comparisons based on 5000 permutations using threshold‐free cluster enhancement (TFCE) scores. All the results are overlaid on a “single‐subject T1” SPM12 image. The scatter plots show the association between each cluster and the mean beta estimates in each cluster; this association is more robust to heterogeneity of response within a cluster (a2, b2, and c2).

Whole‐brain multiple regression analysis revealed that the total schizotypy score was significantly correlated with beta estimates for the “0‐back > rest” contrast in the area from the inferior/middle/superior temporal gyri to the dorsolateral PFC (Figure 3a), in the inferior parietal lobule, the bilateral superior frontal gyri (Figure 3b), in the right middle cingulate cortex (Figure 3c), and in the left superior parietal lobule (Table 7). It is likely that the regions in Figure 3 are associated with task‐induced activation and that higher schizotypy scores are associated with the promotion of task‐induced activation.

**TABLE 7:** Brain regions exhibiting significant interaction effects between schizotypy score and the 0‐back > rest contrast on brain activity.

Brain region	R/L	*X*	*Y*	*Z*	TFCE value	Corrected *p‐*value (FWE)	Cluster size (*k*_E_)
Inferior temporal gyrus	L	−48	−18	−24	1456	< 0.001	8422
Middle temporal gyrus	L	−69	−30	−18	1286	< 0.001	
Superior temporal gyrus	L	−51	−18	−3	1267	< 0.001	
Superior frontal gyrus	L	−21	0	54	795	0.008	96
Superior frontal gyrus	L	−15	12	60	736	0.012	
Inferior temporal gyrus	R	60	−57	−24	757	0.011	13
Superior frontal gyrus	R	21	−9	54	729	0.013	49
Middle cingulate cortex	R	15	−18	48	709	0.014	
Middle cingulate cortex	L	−6	−12	36	711	0.014	37
Superior parietal lobule	L	−24	−63	42	697	0.016	53
Superior parietal lobule	L	−15	−75	45	693	0.016	
Superior parietal lobule	L	−33	−60	54	692	0.016	
Middle cingulate cortex	R/L	0	−21	30	689	0.017	1

Abbreviations: FWE: familywise error, TFCE: threshold‐free cluster enhancement.

*p* < 0.05/3, FWE‐corrected.

**FIGURE 4:**
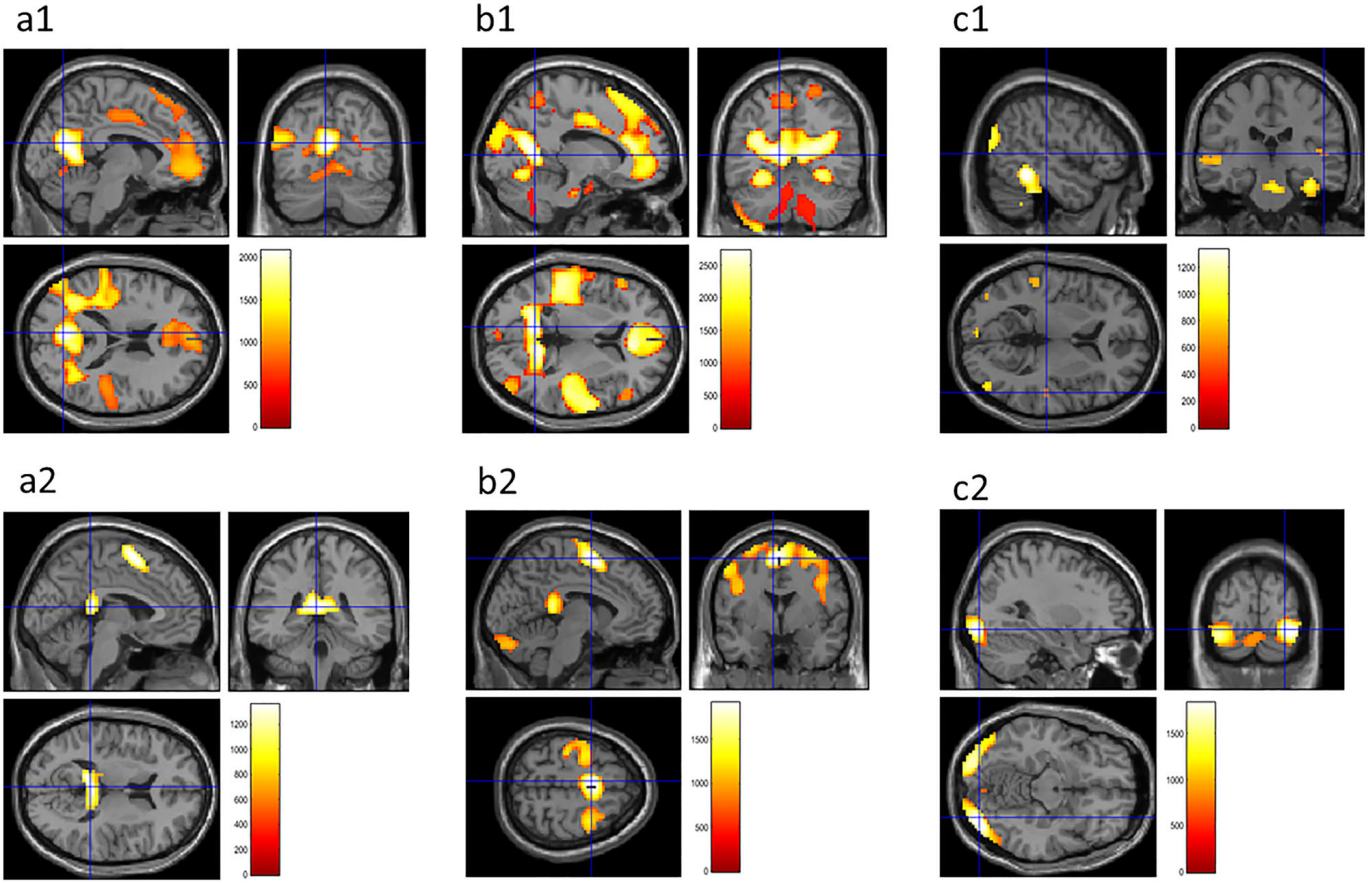
Associations between brain activity during the working memory task and cognitive–perceptual deficits subscores. Cognitive–perceptual deficits subscores were significantly correlated with beta estimates for the “2‐back > 0‐back” (a1), “2‐back < 0‐back” (a2), “2‐back > rest” contrast (b1), “2‐back < rest” (b2), “0‐back > rest” (c1), and “0‐back < rest” (c2) contrast. The results were obtained using a significance threshold of *p* < 0.05/3, corrected for multiple comparisons based on 5000 permutations using threshold‐free cluster enhancement (TFCE) scores. All the results are overlaid on a “single‐subject T1” SPM12 image.

Furthermore, the cognitive–perceptual deficits subscore was significantly correlated with beta estimates for the “0‐back > rest” contrast in the bilateral superior temporal cortex, the left middle occipital gyrus, inferior temporal gyrus, the the fusiform gyrus, the superior frontal cortex, the right inferior temporal gyrus, the precuneus, and the cerebellum (Table 8, Figure 4c1).

**TABLE 8:** Brain regions exhibiting significant interaction effects between the cognitive–perceptual deficits subscore and the 0‐back > rest contrast on brain activity.

Brain region	R/L	*X*	*Y*	*Z*	TFCE value	Corrected *p‐*value (FWE)	Cluster size (*k*_E_)
Inferior temporal gyrus	R	48	−42	−15	1330	< 0.001	965
Middle occipital gyrus	L	−39	−84	21	1157	< 0.001	1122
Fusiform gyrus	L	−27	−57	−15	1012	0.001	795
Superior frontal cortex	L	−21	9	57	798	0.006	68
Superior temporal cortex	L	−51	−18	−3	769	0.007	124
Precuneus	R	24	−60	18	745	0.008	34
Cerebellum	R	18	−54	−42	744	0.008	148
Superior frontal cortex	L	−9	12	66	695	0.012	19
Superior temporal cortex	R	51	−24	6	654	0.016	5

Abbreviations: FWE: familywise error, TFCE: threshold‐free cluster enhancement.

*p* < 0.05/3, FWE‐corrected.

#### 3.2.6 0‐Back < Rest Contrast

No significant positive or negative brain activity related to the total schizotypy score was detected for the “0‐back < rest” contrast via whole‐brain multiple regression analysis.

On the other hand, the cognitive–perceptual deficits subscore was significantly correlated with beta estimates for the “0‐back < rest” contrast in the right occipital gyrus, the left fusiform gyrus, the supplemental motor cortex, the postcentral cortex, and the marginal cortex (Table 9, Figure 4c2).

**TABLE 9:** Brain regions exhibiting significant interaction effects between the cognitive–perceptual deficits subscore and the 0‐back < rest contrast on brain activity.

Brain region	R/L	*X*	*Y*	*Z*	TFCE value	Corrected *p‐*value (FWE)	Cluster size (*k*_E_)
Occipital gyrus	R	30	−87	−12	1831	< 0.001	581
Fusiform gyrus	L	−24	−93	−15	1643	< 0.001	694
Supplementary motor cortex	L	−3	−3	57	1092	0.001	309
Postcentral cortex	L	−42	−24	63	883	0.004	128
Marginal cortex	L	−60	−27	48	668	0.017	1

Abbreviations: FWE: familywise error, TFCE: threshold‐free cluster enhancement.

*p* < 0.05/3, FWE‐corrected.

## 4 Discussion

Our results show that suppression of deactivation of the DMN (the mPFC, the PCC, the inferior parietal lobule, and the middle temporal gyrus) in the working memory task and promotion of its activation (the inferior temporal gyrus and the middle cingulate cortex) during sustained attention are associated with schizotypy scores in a large sample of nonclinical subjects. Among the subscores, only the cognitive–perceptual deficits subscore was significantly correlated with some brain regions. These outcomes are partially consistent with functional changes in the DMN in patients with maladaptive schizotypy and schizophrenia, although consistent patterns have not been observed due to methodological differences (Tonini et al. 2021).

First, we should discuss why the relationship of weaker task‐induced deactivation of the DMN to schizotypy score could be detected not only during the working memory task but also during the sustained attention task. Cognitive control mechanisms are altered during sustained attention in patients with schizophrenia (Hoonakker et al. 2017) and in those with schizotypy (Ettinger et al. 2015). Furthermore, significant activation of the DMN results from brain reorganization, and decreased task‐related suppression in the mPFC is correlated with working memory performance in patients with schizophrenia (Hu et al. 2017). Moreover, reduced suppression of the DMN during working memory tasks has been demonstrated in patients with schizophrenia (Hu et al. 2017; Whitfield‐Gabrieli and Ford 2012). Accordingly, the weaker task‐induced deactivation of the DMN that was observed during the tasks appears to be consistent with disturbance of the DMN in individuals across the schizophrenia spectrum.

Second, we should emphasize that, for the “2‐back > 0‐back” contrast, significant brain activity in the DMN (the medial frontal gyrus, the PCC, and the precuneus gyrus) and the superior temporal gyrus was related not to the total schizotypy score but to the cognitive‒perceptual deficits subscore. In other words, the regions for which associations with schizotypy were identified during the working memory and sustained attention tasks overlapped. In relation to the cognitive–perceptual deficits component, this outcome is consistent with the results of a previous study that showed less deactivation of the left PCC, the superior temporal gyrus, the insula, and the middle frontal gyrus for the 2‐back > 0‐back contrast in the maladaptive schizotypy group than in the healthy control group (Vu et al. 2013). Accordingly, this outcome might be due to differences between the subjects in our study (nonclinical, with attenuated severity) and the clinical subjects who participated in the previous study.

Third, we should explain and discuss the range of suppression of the DMN, that is, the difference between deactivation during the working memory task and activation in the sustained attention task. In healthy people, the degree of task‐induced deactivation increases with task difficulty, for example, with short‐term memory load (McKiernan et al. 2003). The working memory task is more attention‐demanding than the sustained attention task (0‐back task). An inverted U‐shaped relationship between the *n*‐back task load and activity is often observed (Callicott et al. 2003). However, because the average accuracy for the 2‐back task is almost 100%, we might not consider the inverted U‐shaped relationship due to the ceiling effect. Accordingly, it may be natural to detect deactivation during a working memory task and activation during a sustained attention task. Furthermore, siblings of patients with schizophrenia show hyperactivity in the right mPFC, which is a part of the DMN during the encoding phase of working memory (de Leeuw et al. 2013). Together, both the weak deactivation in the regions exhibiting task‐induced deactivation (the DMN) and the strong task‐induced activation might indicate hypersensitivity of the regions.

Fourth, we should focus on the other region outside the DMN, that is, the insula, that showed less deactivation in subjects with schizotypy. On the basis of the findings of relevant sustained attention fMRI studies, the insula appears to be a trait marker for psychosis in patients with schizophrenia (Sepede et al. 2014). Functionally, pathology of the insula, which integrates external sensory input with the limbic system, leads to dysfunction in the processing of visual information in patients with schizophrenia (Wylie and Tregellas 2010). Additionally, volume reduction in the insula may constitute an important neuropathology in schizophrenia patients (Shepherd et al. 2012). Furthermore, the left insula and the precuneus primarily display aberrant activation in first‐episode psychosis; this phenomenon may be associated with attribution of salience to external stimuli and related to deficits in perception and regulation (Soldevila‐Matías et al. 2022). The insula and the anterior cingulate cortex are associated with conflict monitoring under central executive conditions (Wu and Jiang 2020) and during sensory processing (Moon et al. 2021). Accordingly, it appears natural that the insula is affected by differences in schizotypy scores during working memory and sustained attention tasks.

Fifth, it remains unknown why positively and negatively activated brain regions are located in white matter areas of the brain. Importantly, white matter has the vascular capacity to cause hemodynamic changes because venous vessels in white matter are almost the same size as those in gray matter (Gawryluk et al. 2014). Recently, reliable BOLD signals after stimulation have been demonstrated in white matter (Schilling et al. 2022). In a review study in which cortical BOLD responses were modulated by a periodic block design, relevant white matter tracts exhibited corresponding BOLD signals (Mishra et al. 2020). Furthermore, the larger signal change that occurs in white matter was better detected in the 3‐T MRI scan we used than in conventional field strength (1.5 T) MRI (Mazerolle et al. 2013).

Importantly, this study has several limitations. Schizotypal traits in healthy adults are associated with decreased IQ (Matheson and Langdon 2008). However, we used a sample of highly educated young adults (Noguchi et al. 2008). Furthermore, because we excluded subjects with maladaptive clinical schizotypy, the outcomes observed in this study might be based mainly on cognitive‒perceptual deficits. Hence, future studies focusing on the general population may be necessary to determine whether the present findings are generalizable.

## 5 Conclusion

The neural correlates of schizotypy in nonclinical subjects during the performance of working memory and sustained attention tasks are partially coincident with those seen in individuals with schizophrenia spectrum disorders (Barrantes‐Vidal et al. 2015). To clarify the mechanism of preclinical schizotypy, we might focus on the factor of cognitive–perceptual deficits. Further studies using more representative samples are needed to determine whether our results are generalizable.

## Author Contributions

S.N., H.T., Y.T., and R.K. designed the study. S.N., H.T., R.Y., K.S., K.H.d.S.K., T.N., S.Y., T. A, and R.N. collected the data. S.N. and H.T. analyzed the data and prepared the manuscript. All the authors reviewed the manuscript.

## Conflicts of Interest

The authors declare no conflicts of interest.

### Peer Review

The peer review history for this article is available at https://publons.com/publon/10.1002/brb3.70449

## Supporting information



Supporting Information

## Data Availability

The datasets generated during and/or analyzed during the current study are available from the corresponding author on reasonable request.
