# Supplementary material for: Suppression of Deactivation of Working Memory and Promotion of Activation of Sustained Attention in the Default Mode Network Are Affected by Schizotypy in a Large Sample of Nonclinical Subjects
Source: Brain Behav. 2025 May 7;15(5):e70449. doi: 10.1002/brb3.70449 (PMC12056363; doi:10.1002/brb3.70449)
Supplement: Supplementary file 1 — Supporting Information [file BRB3-15-e70449-s001.docx]

Details of psychological measure

The Raven’s Advanced Progressive Matrices (RAPM) (Raven, 1998) described in our previous study (Takeuchi et al., 2022) and the relevant text is reproduced below. The RAPM was used to assess psychometric intelligence. This test contains 36 nonverbal items, which require a fluid reasoning ability. Each item consists of a 3 × 3 matrix with a missing piece to be completed by selecting the best among eight alternatives. The test score, corresponding to the number of correct answers in 30 minutes, was used as an index of individual psychometric intelligence. The test was performed as previously described in a study by our group, using the same assessment methods (Takeuchi et al., 2010). Total score is used in this study as is the case with previous studies (Raven, 2000).

Details of the N-back fMRI task.

Participants received instructions for the tasks and practiced the tasks before entering the MRI scanner. During scanning, they viewed stimuli on a screen via a mirror mounted on a head coil. Visual stimuli were presented using Presentation Software (Neurobehavioral Systems, Inc., Albany, CA, USA). A fiberoptic light-sensitive key press interface with a button box was used to record participants’ task responses. Two conditions were used: 0-back and 2-back. Each condition had six blocks, and all N-back tasks were performed in one session. Subjects were instructed to recall visually presented stimuli (four Japanese vowels) presented “n” stimuli before the currently presented stimulus (e.g., participants had to recall the letter presented two letters earlier for the 2-back task or the currently presented letter for the 0-back task). Two buttons were used during the 0-back task: subjects were instructed to push the first button when the defined target stimuli were presented and the second button when non-target stimuli 6 were presented. During the 2-back task, subjects were instructed to push the first button when the currently presented stimulus and the stimulus presented two stimuli earlier were the same, and to push the second button when the currently presented stimulus and the stimulus presented two stimuli earlier differed. As the four stimuli were presented randomly, the ratio of matched trials to unmatched trials was 1:3 on average. Our version of the N-back task was designed to require individuals to push buttons continuously during the task period. The task level of the memory load was presented above the stimuli for 2 s before the task started and remained visible and unchanged during the task period (cue phase). Each letter stimulus was presented for 0.5 s with a fixation cross presented for 1.5 s between items. Each block consisted of 10 stimuli. Thus, each block lasted 20 s. A baseline fixation cross was presented for 13 s between the last task item and the presentation of the next task level of the memory load (start of the cue phase). Thus, the rest period lasted for 15 s (13 s + 2 s). There were six blocks for each 2- and 0-back condition. The descriptions in this subsection were reproduced from our previous studies of ours from the same project using the same methods (Takeuchi et al., 2020).

Details of image acquisition

For the n-back sessions, 174 functional volumes were obtained. Thorough instructions and thorough fixation by the pad were given as much as possible to prevent head motion during the scan. We did not exclude any subject from the fMRI analyses based on excessive motion during the scan (Takeuchi et al., 2015).

Details of first-level analysis of functional imaging data

The cue phases of the n-back task were modelled in the same manner but were not analysed further. Six parameters obtained by rigid body corrections for head motion were regressed out by adding these variances to the regressor. The design matrix weighted each raw image according to its overall variability to reduce the impact of movement artefacts (Diedrichsen & Shadmehr, 2005). We removed low-frequency fluctuations using a high-pass filter with a cut-off value of 128 s.

References

Diedrichsen, J., & Shadmehr, R. (2005). Detecting and adjusting for artifacts in fMRI time series data. *Neuroimage, 27*(3), 624-634. doi:10.1016/j.neuroimage.2005.04.039

Raven, J. (2000). The Raven's progressive matrices: change and stability over culture and time. *Cogn Psychol, 41*(1), 1-48. doi:10.1006/cogp.1999.0735

Takeuchi, H., Shiota, Y., Yaoi, K., Taki, Y., Nouchi, R., Yokoyama, R., . . . Kawashima, R. (2022). Mercury levels in hair are associated with reduced neurobehavioral performance and altered brain structures in young adults. *Commun Biol, 5*(1), 529. doi:10.1038/s42003-022-03464-z

Takeuchi, H., Taki, Y., Nouchi, R., Yokoyama, R., Kotozaki, Y., Nakagawa, S., . . . Kawashima, R. (2020). Succeeding in deactivating: associations of hair zinc levels with functional and structural neural mechanisms. *Scientific Reports, 10*(1), 12364. doi:10.1038/s41598-020-69277-4

Takeuchi, H., Taki, Y., Sassa, Y., Hashizume, H., Sekiguchi, A., Fukushima, A., & Kawashima, R. (2010). Regional gray matter volume of dopaminergic system associate with creativity: evidence from voxel-based morphometry. *Neuroimage, 51*(2), 578-585. doi:10.1016/j.neuroimage.2010.02.078

Takeuchi, H., Tomita, H., Taki, Y., Kikuchi, Y., Ono, C., Yu, Z., . . . Kawashima, R. (2015). Cognitive and neural correlates of the 5-repeat allele of the dopamine D4 receptor gene in a population lacking the 7-repeat allele. *Neuroimage, 110*, 124-135. doi:10.1016/j.neuroimage.2015.01.053
